# Convolution models for induced electromagnetic responses

**DOI:** 10.1016/j.neuroimage.2012.09.014

**Published:** 2013-01-01

**Authors:** Vladimir Litvak, Ashwani Jha, Guillaume Flandin, Karl Friston

**Affiliations:** The Wellcome Trust Centre for Neuroimaging, UCL Institute of Neurology, London, WC1N 3BG, UK

**Keywords:** EEG, MEG, ERSP, Induced responses, Time–frequency analysis, General linear model, Convolution, Statistical parametric mapping

## Abstract

In Kilner et al. [Kilner, J.M., Kiebel, S.J., Friston, K.J., 2005. Applications of random field theory to electrophysiology. Neurosci. Lett. 374, 174–178.] we described a fairly general analysis of induced responses—in electromagnetic brain signals—using the summary statistic approach and statistical parametric mapping. This involves localising induced responses—in peristimulus time and frequency—by testing for effects in time–frequency images that summarise the response of each subject to each trial type. Conventionally, these time–frequency summaries are estimated using *post‐hoc* averaging of epoched data. However, *post‐hoc* averaging of this sort fails when the induced responses overlap or when there are multiple response components that have variable timing within each trial (for example stimulus and response components associated with different reaction times). In these situations, it is advantageous to estimate response components using a convolution model of the sort that is standard in the analysis of fMRI time series. In this paper, we describe one such approach, based upon ordinary least squares deconvolution of induced responses to input functions encoding the onset of different components within each trial. There are a number of fundamental advantages to this approach: for example; (i) one can disambiguate induced responses to stimulus onsets and variably timed responses; (ii) one can test for the modulation of induced responses—over peristimulus time and frequency—by parametric experimental factors and (iii) one can gracefully handle confounds—such as slow drifts in power—by including them in the model. In what follows, we consider optimal forms for convolution models of induced responses, in terms of impulse response basis function sets and illustrate the utility of deconvolution estimators using simulated and real MEG data.

## Highlights

► We propose a new approach to analysis of induced responses in M/EEG. ► The General Linear Model is used to model continuous power as in fMRI 1st-level. ► The results can be presented as conventional time–frequency images. ► Our method is better for experiments with variable timing and overlapping events.

## Introduction

Time–frequency analysis is a widely used method for studying induced neural responses, particularly in terms of oscillatory brain activity. The standard procedure for time–frequency analyses involves selecting data segments around events of interests (epochs) and subjecting them to spectral analysis using a time-resolved method (e.g. wavelet analysis (Tallon-Baudry et al., 1997), Hilbert transform (Swettenham et al., 2009), windowed Fourier analysis (Mitra and Pesaran, 1999)). This creates a two-dimensional image of spectral power—for each epoch—over time and frequency. These time–frequency images are then averaged, possibly transformed or baseline-corrected to emphasise particular data features and subjected to statistical analysis (Kilner et al., 2005; Maris and Oostenveld, 2007). This *post‐hoc* trial averaging procedure is analogous to the analysis of evoked responses in the time domain. While this approach has proved its usefulness in numerous studies, it also has several limitations.

Unlike evoked responses—that decay within about half a second to a second—induced changes in power can last for several seconds or even longer (with sustained stimuli). Consequently, in many paradigms whose trials comprise multiple events, the responses to different events overlap and confound each other—making it difficult to characterise them separately. Another problem arises when the paradigm involves subject responses, which introduce an inevitable variability in reaction times and associated neuronal responses (Gibbons and Stahl, 2007; Yin and Zhang, 2011). This makes it difficult to interpret the average (induced response) because a particular peristimulus time in different trials may correspond to different stages of neural processing. One possible solution—used in the literature for both time-domain and frequency-domain data—is to sort trial-specific responses by reaction time (Makeig et al., 2004). However, these sorted responses are not pooled in any subsequent statistical analysis (e.g. to test for a difference between two experimental conditions, while modelling the between-trial differences in timing). Variable latencies can also confound statistical analysis of experimental effects: if two conditions differ systematically in the reaction time (e.g. trials with valid and invalid cues in the Posner task) there will be systematic differences in the corresponding time–frequency activity. *Post‐hoc* averaging procedures are unable to distinguish true differences in induced power from reaction time confounds—we will refer to this as the latency confound problem. Furthermore, when baseline correction is used as part of the analysis, systematic differences in the baseline between conditions may also confound the results. Finally, in naturalistic tasks (e.g. continuous navigation in virtual reality environment) it might be difficult to define discrete epochs over which to take trial averages.

Here we propose a new framework for the analysis of time–frequency data that finesses these problems. This framework is based on the well-established methodology for statistical analysis of fMRI time series: in brief, we dispense with *post-hoc* trial averaging of epoched data and model the continuous time series of induced responses (over multiple frequencies) using a linear convolution model. This allows one to encode specific stimuli or response events as experimental input functions and model the observed induced responses in terms of induced response functions. Not only does this allow for the superposition of multiple induced responses to different events occurring in close temporal proximity—but it also allows for a parametric modulation of the amplitude of induced responses from trial to trial.

This paper is organised as follows. In the first section we briefly recap the main principles of general linear model (GLM)-based analyses and show how they can be applied to continuous time–frequency data. In the second, we establish face validity of the approach using simulated time–frequency power data and discuss the conditions under which it is optimal. In the third section, we demonstrate the method using real data by applying it to a magnetoencephalography (MEG) data acquired from a subject performing the change-of-plan task. These data present particular challenges that include trial to trial variations in the temporal composition of induced response components. We hope to show that the new convolution model meets these challenges.

## Methods and theory

The linear convolution model for analysis of neuroimaging time series was introduced by Friston et al. (1994) for the analysis of PET and fMRI data (Boynton et al., 2012; Poline and Brett, 2012). The basic idea is to model the time series from each voxel with a linear mixture of independent variables (regressors) represented by columns of a design matrix. In the case of fMRI time series these regressors are created by convolving input functions encoding the experimental design (e.g. a series of delta functions representing stimulus presentations) with basis functions capable of efficiently representing the shape of the haemodynamic response (Josephs and Henson, 1999). The most commonly used basis set is the canonical hemodynamic response function (HRF) and its partial derivatives with respect to time and dispersion. The parameters of the ensuing general linear model can then be applied to the basis set to model the impulse response function to each stimulus or response event of interest. Furthermore, images of the parameter estimates can be subjected to statistical parametric mapping (SPM) to make classical inferences about regionally specific responses in the usual way. The haemodynamic basis set is specifically tuned for fMRI responses and would therefore be suboptimal for M/EEG time–frequency responses. One could derive a new canonical basis set specific to M/EEG (Ramkumar et al., 2010). However, there are several generic basis sets that could be used irrespective of the data modality. For example, the basis sets used for fMRI time series include:

• Finite impulse response (FIR) basis set—a series of rectangular functions that tile peristimulus time.
• Fourier set—a set of sine and cosine functions of peristimulus time.
• Fourier set multiplied by the Hanning taper—similar to Fourier set but better for modelling responses that are concentrated in the centre of the analysis time window.
• Gamma functions—a mixture of functions suitable for most transient responses.

All these basis sets are capable—in principle—of modelling transient induced power responses and we will demonstrate below how the best basis set can be selected and optimised for a particular application.

Another difference between fMRI and time–frequency analysis lies in the interpretations of the results. In fMRI, the time course of the BOLD response is not of interest in most cases. Therefore, the estimated parameters or their linear combinations (contrasts) can be tested directly to identify significant responses at particular locations in the brain. In the analysis of induced responses one is generally interested in the testing for responses at particular locations in peristimulus time and frequency. The approach proposed here uses a linear convolution model to estimate (the parameters of) impulse response functions specific for each event type and uses these to summarise the induced response (over frequencies) for these event types—as if each of them had been presented in isolation. These reconstructed responses take the form of conventional time–frequency images that can be analysed in exactly the same way as conventional *post-hoc* trial averages.

### Building the convolution model

**Fig. 1:**
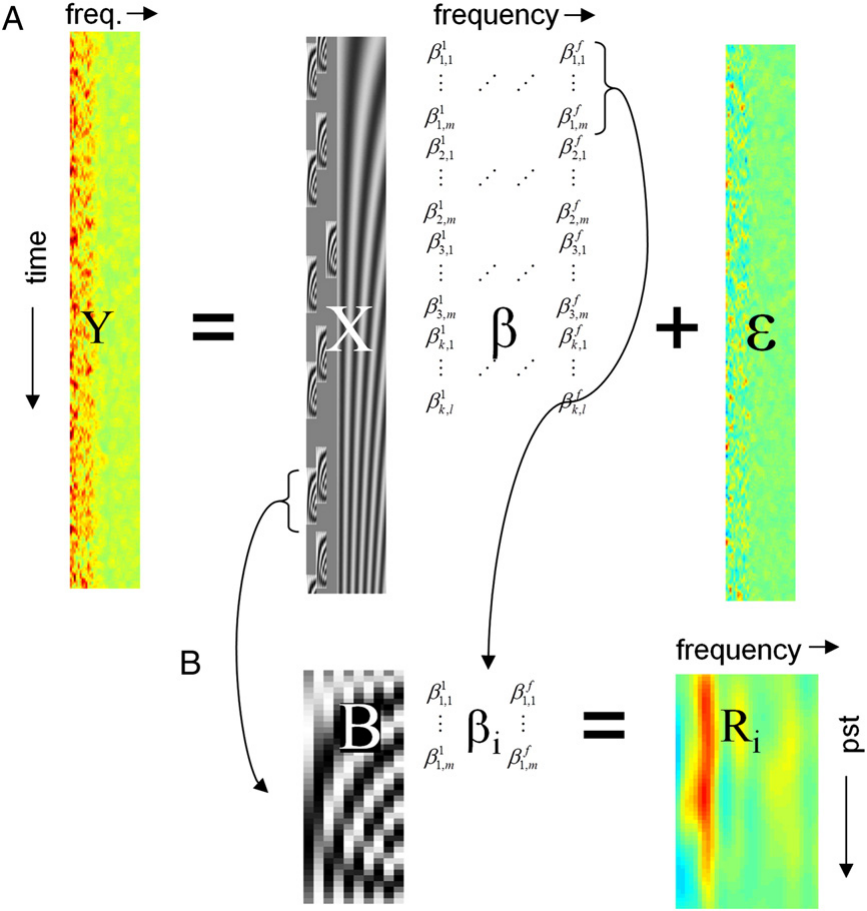
Schematic representation of the GLM approach for the analysis of time–frequency data. A. Continuous time–frequency recording Y is modelled as the product of design matrix X and coefficients β with additive noise ε. X contains basis functions for each event and regressors modelling confounds (e.g. slow drifts). The GLM coefficients are estimated using ordinary or weighted least squares. B. Event-type specific time–frequency images R_i_ are reconstructed by multiplying β_i_—the GLM coefficients corresponding to the i-th event type—by the basis set B. These correspond to a least squares deconvolution of event-related responses from the original time-series.

The general linear model explains the response variable Y in terms of a linear combination of explanatory variables and noise (Fig. 1A).

(1) Y=X⋅β+ε

Here, $Y\in R^{t\times f}$is the matrix of continuous power with *t* time bins and *f* frequency bins, $X\in R^{t\times n}$ is the design matrix whose n columns contain explanatory variables or regressors that can be combined linearly to explain the observed time series of induced power and $\varepsilon \in R^{t\times f}$is the noise matrix such that *ε* ~ *N*(0,*C*), where $C\in R^{t\times t}$ is the covariance matrix of the noise. The parameters of this model $\beta \in R^{n\times f}$ constitute a matrix of unknown regression coefficients for each regressor and frequency to be estimated.

Given the diversity of possible impulse response functions for induced responses, we considered a generic basis set capable of representing any physiological response shape: induced responses cannot be very fast for physiological reasons (M/EEG signals are generated by the summation of a large number of post-synaptic currents that necessarily involves some temporal smoothing) and computational reasons (any spectral estimation method has limited time resolution due to the uncertainty principle). We construct regressors by convolving a set of basis functions with a set of input functions representing the events of interest (e.g. delta or stick functions for each stimulus or boxcar functions for experimental manipulations that persist over time). For *m* basis functions $B\in R^{p\times m}$ over *p* peristimulus time bins and *k* input functions $U\in R^{t\times k}$ this results in *n* = *m* × *k* regressors, where one column of the design matrix, $X_{\left(i-1\right)\cdot m+j}=U_{i}*B_{j}\in R^{t\times 1}$_,_ is the regressor for the *i*-th event and *j*-th basis function.

### Summarising induced responses

Following the estimation of GLM coefficients *β* by ordinary or weighted least squares, the impulse response function for a particular event type and frequency can be reconstructed by multiplying the basis functions with the parameter estimates corresponding to the event type and frequency in question. When this is repeated for all frequencies—and the same event type—the ensuing response functions of peristimulus time constitute a time–frequency image: *R*_*i*_ = *B* ⋅ *β*_*i*_, where $\beta_{i}\in R^{m\times f}$ corresponds to the parameter estimates for the *i*-th event type over frequencies (Fig. 1B). This $R_{i}\in R^{p\times f}$ response image can be interpreted as a deconvolved time–frequency response to the event which would be seen (under linear assumptions) if the event was presented in isolation. These time–frequency images summarise the induced responses of each subject to a particular event type. They can then be subjected to standard SPM analysis at the second (between-subjects) level in the usual way (Kilner et al., 2005).

### Extensions of the approach

#### Drifts and baseline correction

The GLM framework makes it possible to introduce additional regressors for modelling data components of no interest; e.g., slow drifts, eye blinks or movements in the MEG scanner. If computational constraints allow, power series from several recording sessions can be concatenated and modelled together. In this case, a separate set of slow drift regressors can be added for each session—to handle discontinuities. Note that these drift regressors play the same role as baseline correction in conventional *post-hoc* averaging. However, in the setting of a convolution model, this baseline correction is informed by fluctuations in the data over all time points.

#### Modelling parametric effects

In addition to modelling the average effect of an event on induced power, modulations of this effect by experimental factors can be modelled using parametric modulators—a method well established in fMRI time series analysis. This involves adding to the basic set of regressors—modelling the average response—one or more additional sets where the input functions encoding events are scaled in a trial specific fashion according to some parametric experimental factor—for example, the monetary value of a stimulus or some variable attribute of a motor response such as force. The corresponding time–frequency response image *R*_*i*_ now reflects a modulation of induced responses by the parametric factor, which can be tested for using the same SPM procedures that are used to test for average or mean responses.

#### Continuous regressors

For the examples in the present paper, we generated regressors by convolving basis set functions with series of delta functions encoding the times of occurrence of experimental events—usually called stimulus functions. However, it would also be possible to use more complicated stimulus functions to form regressors. The simplest example is a boxcar function, for experimental manipulations that persist over time (as mentioned above); however, one could also use a continuous variable (for example, the velocity of a moving grating in a visual experiment). In this case, the interpretation of the resulting time–frequency image would be parametric—namely, the change in power per unit change in the experimental attribute encoded by the parameter. In other words, it would represent the impulse response function for the corresponding stimulus attribute rather than average response induced by stimulus.

#### Application to data in the time domain (evoked responses)

Although we have focused on an application of the convolution method to induced power, in principle, the same approach could also be applied to evoked responses—where the main advantage would be to separate responses to overlapping events and remove latency confounds. In that case one would not model multiple frequencies but just reconstruct a single time course by combining the regressors as is standard for fMRI. The main problem with convolution models of continuous data—in the time domain—is the high computational cost. The typical number of samples in fMRI time series is around one thousand. The power time series such as used in this paper contain thousands to tens of thousands of samples and continuous data in the time domain would comprise tens to hundreds of thousands of samples. Given the faster dissipation of evoked (compared to induced) responses, in most cases the use of GLM would not be justified but one could certainly envision its application where necessary.

#### Suppressing artefacts

Contamination by artefacts is common in M/EEG. These artefacts might be extended in time. Even when they are short, several consecutive samples in power time series might be affected because the time windows for estimating spectra are extended in time and can overlap. This is different from the situation in fMRI where artefacts usually affect an isolated volume in a series which can be excluded by adding a regressor modelling out the signal from the corresponding time point. The GLM framework offers a way of suppressing the influence of artefacts while still modelling continuous power time series. This can be done by using the weighted least squares approach where the residuals at the parts of the time series affected by artefacts are assumed to have very high variance. Computing the optimal estimate of the weights in this case involves pre-whitening of the data and the design by the square root of the inverse of the covariance matrix which effectively suppresses the artefact-contaminated parts.

#### Optimal convolution models and experimental design

To estimate the parameters of the convolution model efficiently the regressors (columns of the design matrix) should not be highly correlated. When using an orthogonal basis set, regressors pertaining to the same event will always be orthogonal by design. However, regressors pertaining to different events might be correlated. The reasons for this are intuitive: if two events always occur together with the same latency, it is impossible to attribute induced responses uniquely to either event. Variability in the latency between events—across repetitions—can render the design less correlated (more efficient), but endogenous variability (such as that of reaction times) might not be sufficient to ensure an efficient design. It is therefore necessary to optimise the experimental design with efficiency in mind. This is standard practice in fMRI design and usually involves paradigms in which trials do and do not contain a component (event) of interest (Josephs and Henson, 1999). For instance, if one wanted to disambiguate responses to a stimulus and subsequent motor response, it would be necessary to have trials with and without motor responses—by either delaying the motor response in some trials or omitting it completely using no-go trials. The advantage of modelling induced responses with a continuous convolution model means that one can exploit designed and endogenous differences between trials to isolate response components in a way that is not possible using *post-hoc* averaging.

### Summary

In this section, we have described a summary statistic approach to the analysis of induced response components. This approach rests on summarising response components (or their parametric modulation by an experimental factor) in terms of an impulse response function to designed or measured experimental perturbations. These time–frequency summaries are obtained from a least-squares deconvolution, using a relatively simple general linear model, in which the impulse response function comprises a mixture of orthogonal basis functions. Having estimated the maximum likelihood time–frequency response for trial components of interest, these are analysed in the usual way—using statistical parametric mapping to identify significant responses that are localised in time and frequency (Kilner et al., 2005).

As noted above, summary responses are produced using, essentially, ordinary least squares de convolution. Since the time–frequency data is inherently correlated over time, one might argue a weighted least squares (maximum likelihood) scheme would be more appropriate. Although this is true—in the application considered here—inference is performed at the second (between-subject) level and the issue of serial correlations at the first (within-subject) level is largely irrelevant. This is because the serial correlations at the first level do not bias estimates of the de-convolved response—they only bias estimates of their standard error. These standard errors are not passed to subsequent levels for inference in the summary statistic approach. One could get more efficient first level (maximum likelihood) estimates using a weighted least squares scheme; however, this would incur some computational cost—which we considered prohibitive in this particular setting.

In the next section, we examine the face validity and efficiency of this approach using synthetic data.

## Simulations

In this section, we examine the performance of the proposed procedure using simulated power time series. The simulated responses were restricted to the beta band. The beta signal was generated by band-pass filtering Gaussian white noise with amplitude of 1 arbitrary units (a.u.) between 15 and 35 Hz. Two kinds of responses were simulated—Event-Related Synchronisation (ERS) modelled with an increase in signal amplitude of 100% (unless indicated otherwise) and Event-Related Desynchronisation (ERD) modelled with a decrease in signal amplitude of 90%.^1^ The ERS can be considered as induced by a stimulus and the ERD—by subject responses. The time courses of both induced responses were Gaussian bump functions with a standard deviation of 125 ms for the ERS and 250 ms for the ERD. ERS occurred at fixed intervals of 5 s. ERD events occurred around the ERS events in half of the trials (selected at random). The ERS–ERD latency distribution was varied over different simulations (see below for details). The total duration of each simulated response was 450 s. To generate the final signal we combined the ERS and ERD in one of two possible ways.

**Fig. 2:**
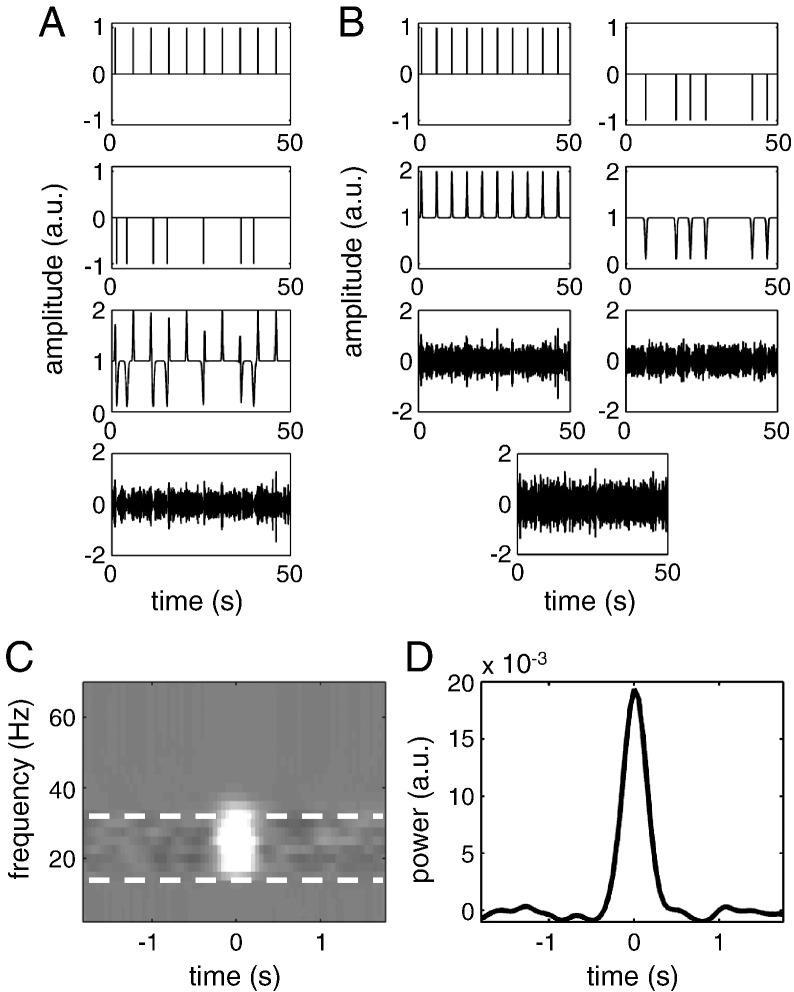
Simulation setup. A. Source-level mixing. Only a short 50 s segment out of total of 450 s is shown. From top to bottom: impulses corresponding to ERS events, impulses corresponding to ERD events, combined amplitude waveform, the final signal consisting of band-filtered noise modulated by the amplitude waveform. B. Similarly for sensor-level mixing. From top to bottom: impulses corresponding to events in the two sources (ERS on the left, ERD on the right), corresponding amplitude waveforms, source signal generated by modulating two different instantiations of band-filtered noises by the amplitude waveforms, the final sensor-level signal created by summation of the source-level signals. C. Time–frequency image created for the source-level simulation where only ERS events were present with the conventional epoching approach with baseline correction. Note that the signal is mostly concentrated in 15–30 Hz band (indicated by the dashed white lines) and around the time of zero there is an increase in power. D. Power time course obtained by summing the image shown in (C) over the 15–30 Hz band. This is an example of the kind of waveforms shown in Figs. 3–6.

• Source-level mixing modelled a scenario where the same neuronal population expresses both synchronisation and desynchronisation (at different latencies). In this case, an amplitude modulation waveform was created by adding the ERS and ERD to a baseline level of unity. The amplitude modulation was then applied to the filtered synthetic beta time series (Fig. 2A).
• Sensor-level mixing modelled a scenario in which ERS and ERD occurs in distinct neuronal populations and mixing occurs at the sensor level. To model this situation, we generated two separate beta time series—one with ERS and the other with ERD. Each of the beta time series were modulated separately, after which the two signals were combined (Fig. 2B).

The simulations were repeated with and without adding white Gaussian noise—with an amplitude of 1 a.u.—to the modulated beta signal. White noise was chosen as the simplest option. Using noise with a more realistic spectral profile (e.g. 1/f noise) would not make much difference since we analyse each narrow frequency band independently.

### Time–frequency analysis

The simulated time series signal was analysed with both standard epoching and *post-hoc* averaging and deconvolution using SPM8 toolbox (Litvak et al., 2011) supplemented by custom MATLAB code. For the standard analysis, the continuous signal was epoched separately around episodes of ERS and ERD from 2 s before to 2 s after the response.

Time–frequency analysis was performed on either epoched or continuous data. We used a multitaper spectral analysis (Thomson 1982)—but other spectral estimation methods could also be used with the GLM approach. The time window of 0.4 s was shifted in steps of 0.05 s. Power was estimated for frequencies from 2.5 to 90 Hz in steps of 2.5 Hz—although for simulated data only the results for 15–30 Hz range were actually examined. The frequency resolution was set to the inverse of the time window (2.5 Hz) for up to 25 Hz, then 0.1 times the frequency for 25 to 50 Hz and then to constant 5 Hz. These settings mean a single taper was used for 2.5–30 Hz, 2 tapers for 32.5–42.5 Hz and 3 tapers for 45 Hz and above. The resulting time–frequency images had no discontinuities in frequency due to the continuous frequency resolution function.

Following spectral estimation, it is a common practice to transform power data (e.g. to render the noise components approximately normal (Kiebel et al., 2005)). We compared untransformed power data to those transformed with log and square root transform to find the transform that optimised the performance (sensitivity) of our convolution method.

For standard analyses the time–frequency data were averaged over epochs and baseline-corrected (baseline − 2 to − 1.5 s relative to the event). For the deconvolution analysis, we included a discrete cosine set with frequencies up to 0.1 Hz in the design matrix to remove (baseline) drifts. Time–frequency images were generated as described above using input functions encoding ERS and ERD peaks convolved with a Fourier basis set of order 11 (comprising of 22 basis functions, 11 sines and 11 cosines). The selection of a basis set is considered later (in “Optimising the basis set”). Given the nature of our simulated data, there were no frequency-specific responses, other than in the beta band. Therefore we converted the time–frequency images into waveforms by averaging across the 15–35 Hz range (Figs. 2C and D) to simplify the presentation of the results.

### Results

#### Effect of transform and variability in ERS–ERD latency

**Fig. 3:**
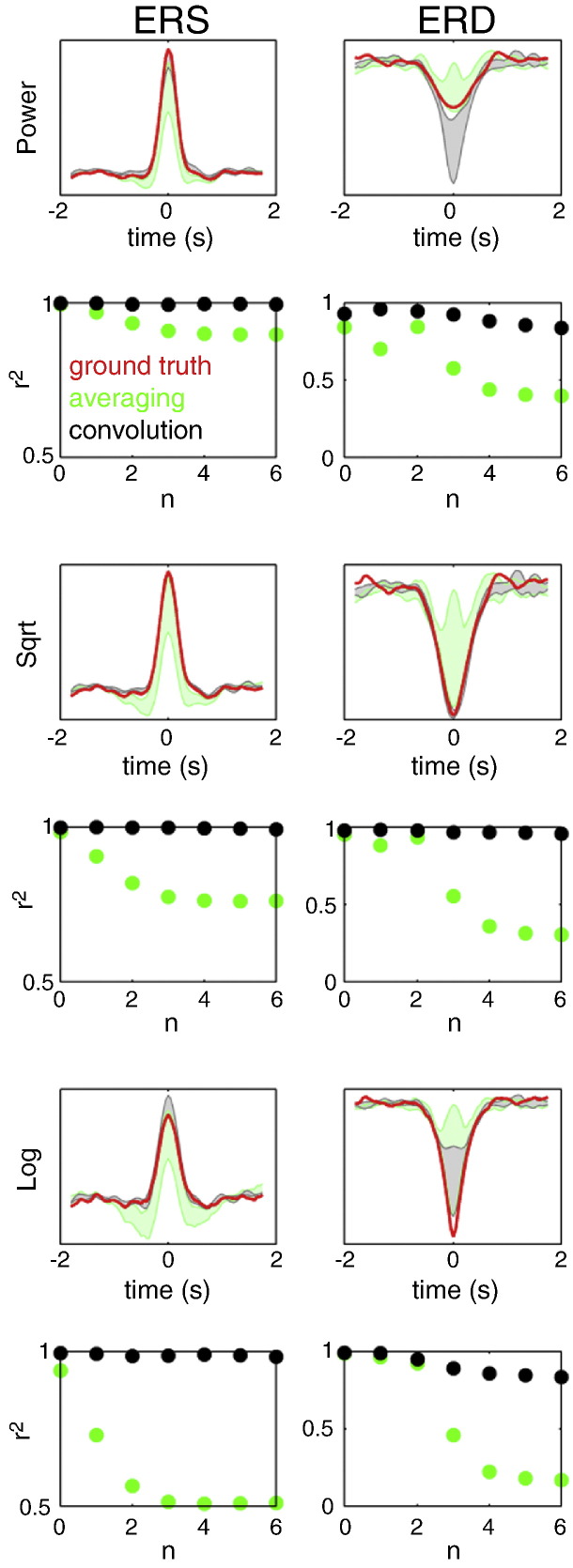
The results of source-level mixing simulations comparing the epoching and convolution approaches and three power rescaling methods. The mean latency between ERS and ERD was set to zero with a standard deviation of the form 2^− *n*^ s where *n* = [0,1,…,6]. The estimated power responses for the epoching method (green) and for the convolution method (black) were compared to ground truth waveforms, obtained by simulating ERS and ERD separately (without noise) and performing time–frequency analysis using the epoching approach with baseline correction (red). Three power rescaling methods were tested: no rescaling (top two rows), square root (middle two rows) and log (bottom two rows). To simplify the figures we do not show the individual traces for the different values of n but just the areas between the minima and maxima for all traces. The bottom row for each rescaling method shows the coefficient of determination (r^2^) for correlations between each of the individual reconstructed power time courses and the ground truth time course. Note that combining square root rescaling with the convolution approach makes it possible to recover the simulated waveforms accurately for all latency distributions tested—and that the convolution method consistently outperforms averaging.

**Fig. 4:**
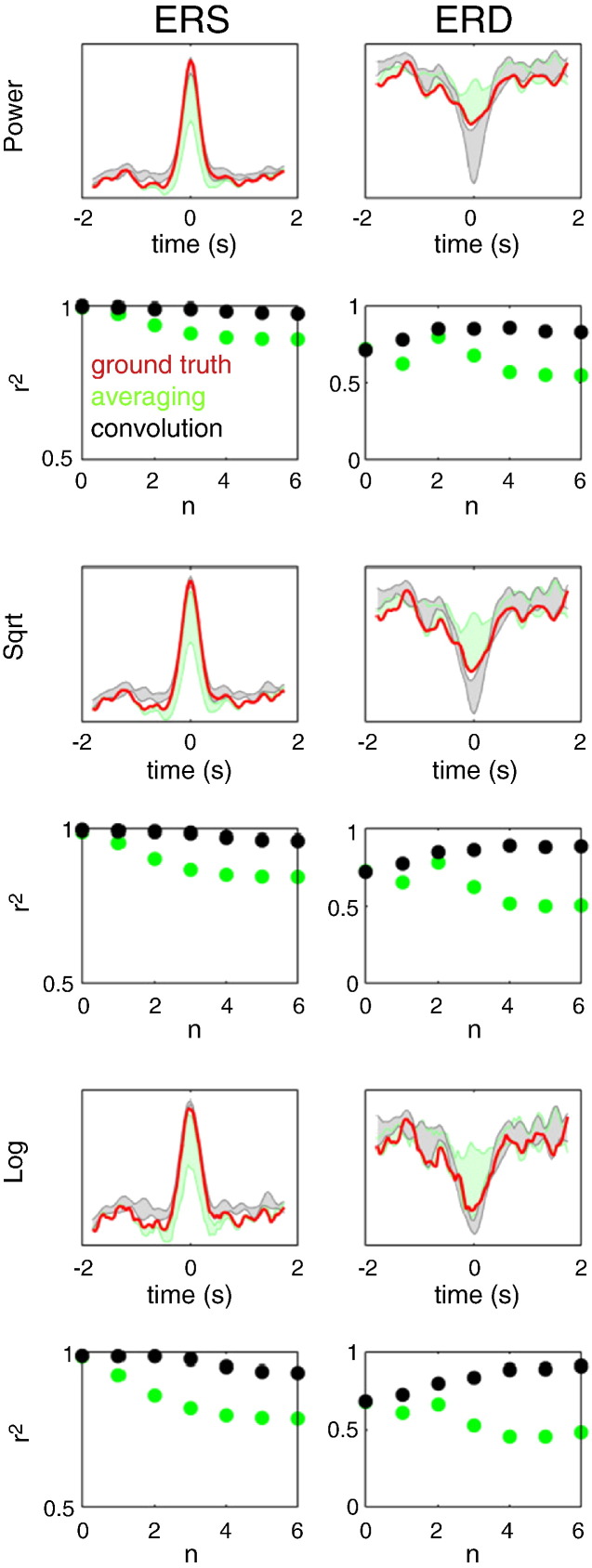
The results of source-level mixing simulations with added Gaussian white noise (SNR = 1). The simulations were similar to those shown in Fig. 3. Note that the convolution method with either log or square root transform provides good approximations to the ground truth, under relatively high levels of noise.

In this series of simulations, the mean latency between ERS and ERD was set to zero with a standard deviation of the form 2^− *n*^ s where *n* = [0,1,…,6]. The estimated power responses were compared to ground truth waveforms obtained by simulating ERS and ERD separately (without noise) and performing time–frequency analysis using the standard approach with baseline correction. The results for source-level mixing are shown in Fig. 3. The goodness-of-fit between the reconstructed power time courses and the ground truth was quantified by the coefficient of determination (r^2^). When using the standard approach the estimated power waveforms approximated the ground truth only for the largest latency variability (std = 1 s). When the variability decreased, ERS and ERD could not be separated and the power waveforms were mixtures of the two. In contrast, the GLM method—with square root transform—performed well for all latency distributions. Note that a major factor for this superior performance was the fact that the effects of ERS and ERD were decorrelated by omitting the ERD in half of the trials. This is something that *post-hoc* averaging cannot exploit, especially for ERD that always occurs in the presence of ERS. Fig. 4 shows the results of similar simulations with Gaussian white noise (SNR = 1). One can see that the GLM method still performs well. Both log and square root transforms seem to provide good approximations to the ground truth, under relatively high levels of noise.

**Fig. 5:**
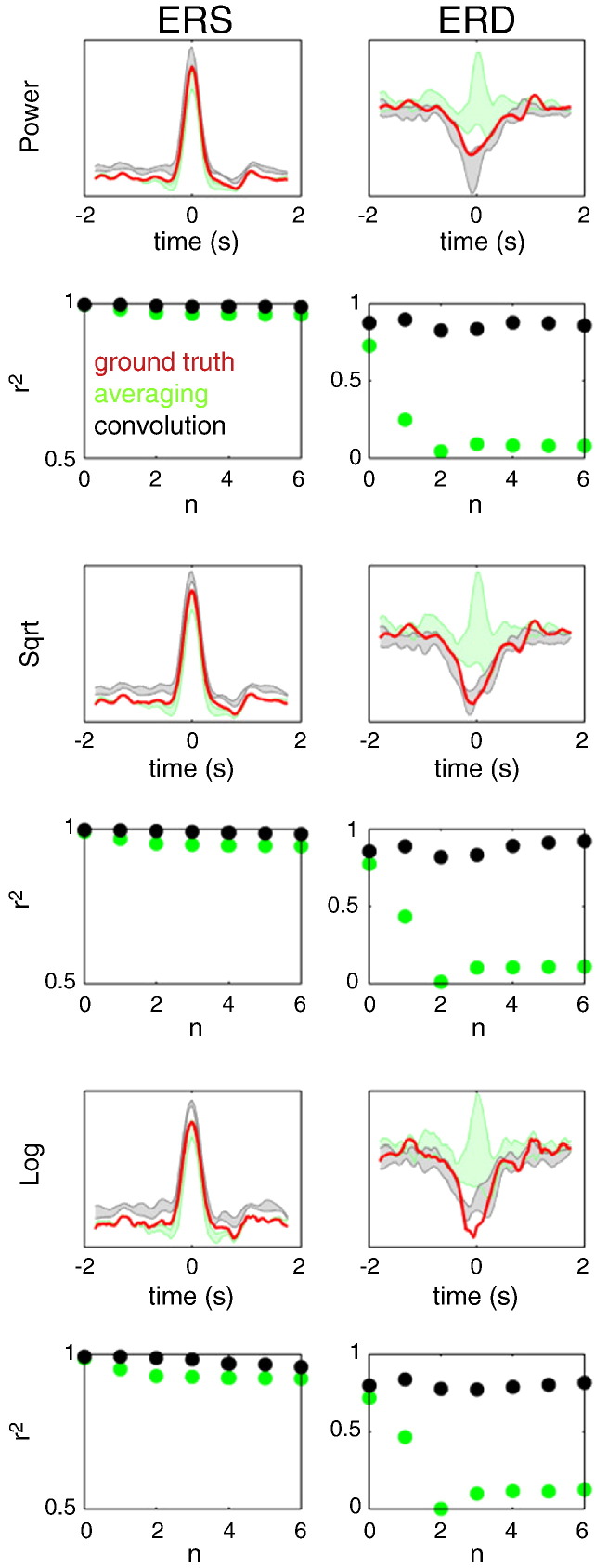
The results of simulations with sensor-level mixing and no noise. Here again, the GLM method is capable of separating ERS from ERD responses—with a square root transform giving the best results.

Fig. 5 shows the results of simulations with sensor-level mixing and no noise. Here again, the GLM method is capable of separating ERS from ERD responses—with a square root transform giving the best results. Note that recovery of the ERD time course is more difficult here than in the source-level mixing case because—even in the absence of simulated noise—because there is beta band activity from the ERS source.

#### Artefact suppression

**Fig. 6:**
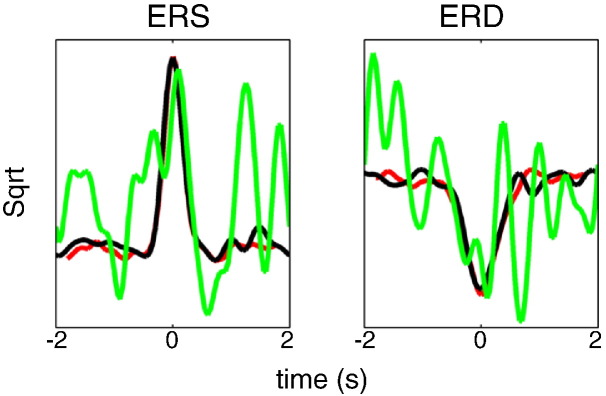
Artefact suppression using the weighted least squares method. The simulated signal was contaminated by 100 randomly placed spikes with amplitude of 100 a.u. GLM analysis was performed without (green) and with (black) artefact suppression using the weighted least squares method. The red waveform shows the ground truth as in Fig. 3.

To demonstrate artefact suppression with the weighted least squares approach we added to the simulated time series one hundred randomly positioned spikes with the amplitude of 100 a.u. (compared with 1 a.u. standard deviation of the simulated signal). The large amplitude of the artefacts was necessary to clearly see their effect in the absence of artefact suppression, as the ordinary least squares GLM method was robust to artefacts of smaller amplitude. We then detected the artefacts by thresholding the time domain signal at 2 a.u. Based on our knowledge about the resolution of the time–frequency decomposition we set the elements on the diagonal of the weighting matrix W, corresponding to time bins that could be affected by the artefacts to 2^– 256^ (effectively zero). Fig. 6 shows the results of model estimation using this approach compared to ordinary least squares. The weighted least squares method makes it possible to completely recover the simulated induced response in the presence of artefacts.

#### Removing the latency confound

**Fig. 7:**
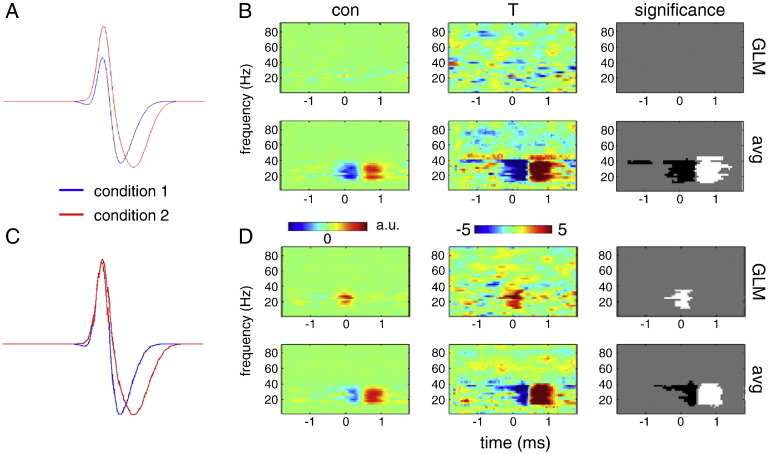
Removing the latency confound. Simulations were performed where the trials were divided into two conditions. In condition 1, the mean latency between ERS and ERD was 0.3 s and in condition 2 it was 0.6 s. In both conditions the latencies were drawn from a normal distribution with standard deviation of 0.125 s. A. Average amplitude waveforms for the two conditions, time-locked to the ERS. Note that the apparent amplitude of the ERS is reduced in condition 1 due to greater overlap with the ERD—although in reality the amplitude of ERS is equal in both conditions. B. Results of two-sample t-test between time–frequency images for the ERS of condition 1 and condition 2 performed across 10 repetitions of the simulation. Time–frequency images were computed using GLM (top row) or averaging (bottom row). C. Average amplitude waveforms for the simulation where the amplitude of the ERS in condition 1 was adjusted to equate peak average ERS amplitudes for the two conditions. D. Results of two-sample t-test between time–frequency images for the ERS of condition 1 and condition 2 performed across 10 repetitions of the simulation with adjusted ERS amplitude.

One of the major advantages of the convolution method is that it can resolve the confounding effect of latency differences, when estimating the amplitude of condition-specific induced responses. To illustrate this problem, simulations were performed where the trials were divided into two conditions. In condition 1, the mean latency between ERS and ERD was 0.3 s and in condition 2 it was 0.6 s. In both conditions the latencies were drawn from a normal distribution with standard deviation of 0.125 s. Fig. 7A shows the average amplitude waveforms for the two conditions, time-locked to the ERS. It can be seen that the apparent amplitude of the ERS is reduced in condition 1 due to greater overlap with the ERD—although in reality the amplitude of ERS is equal in both conditions. Fig. 7B shows the results of two-sample t-test between time–frequency images for the ERS of condition 1 and condition 2 performed across 10 repetitions of the simulation. *Post-hoc* averaging, produced (falsely) significant differences around zero (the peak of the ERS) and later around the time of the ERD. In contrast, the GLM method produced no significant differences and furthermore the image of the difference between two ERS estimates shows little structure. To demonstrate that this is not due to a lack of power of the GLM method, we adjusted the ERS amplitude in condition 1 so that the average amplitudes around zero were equivalent in the two conditions (Fig. 7C). Here, there was no apparent difference in the peak amplitude of the ERS because it is obscured by differences in reaction time. Statistical analysis using the GLM method reveals a significant difference in the ERS amplitude around zero (Fig. 7D). In contrast, *post-hoc* averaging only reveals significant differences at later times—that reflect the reaction time effects, rather than true differences in power.

#### Optimising the basis set

As described above, the optimal basis set suitable for the convolution model comprises a small number of functions that can fit accurately the form of induced power responses observable in the data. In this paper, we used a Fourier basis set, but one could envision other basis sets—perhaps specifically optimised for the power responses that are generated by neural masses. Here we describe some procedures for comparing such basis sets and determining the optimal number of basis functions. These optimisation methods are not new; we just reiterate them here in the context of our new application.

**Fig. 8:**
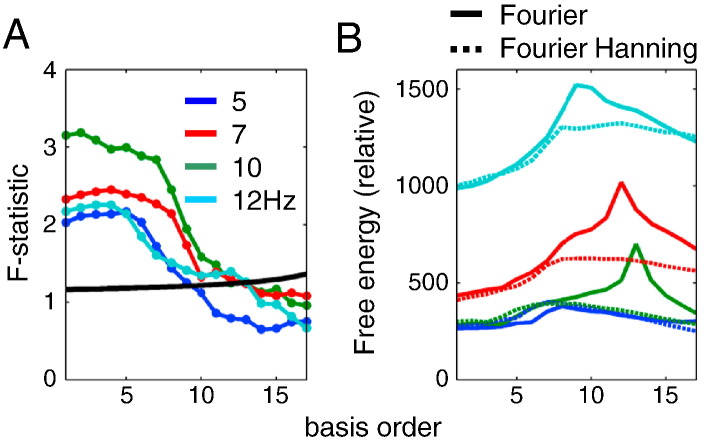
Optimising the basis set for the GLM analysis. We used real data from a change-of-plan experiment (see the main text) to compare different basis sets for the GLM analysis. The different curves represent different frequencies: 5 Hz (blue), 7 Hz (red), 10 Hz (green) and 12 Hz (cyan)—as the results can vary depending on empirical responses. A. Optimising the number of basis functions in the Fourier set using F-test. The black curve shows the threshold of significance at *p* = 0.05. Note that the threshold increases when adding regressors (basis function components) to the design matrix, due to the implicit change in the degrees of freedom. The optimal number here is the highest number above the threshold line (between 9 and 13 Hz depending on frequency). B. Comparing non-nested basis sets, Fourier (solid lines) and Fourier Hanning (dotted lines) using Parametric Empirical Bayes. Here the optimal basis set is the one with the peak model evidence (approximated by free energy). For each frequency we subtracted the minimal free energy value from the other values. For all frequencies except 5 Hz the Fourier basis set had higher evidence and the optimal number of basis functions was between 8 and 13 consistent with the F-test results.

One can determine the optimal number of functions in a given basis set using the extra sum of squares principle or F-tests. This procedure tests whether the proportion of variance explained by a subset of regressors in the design matrix is significant, with respect to unexplained variance. One can start with a single basis function and then increase the number of basis function components—testing at each step whether the extra basis functions explain significant additional variance. Fig. 8A shows an example of this (step-up) procedure applied to real MEG data—from the change-of‐plan experiment described in the final section. We examined data from four different frequencies: 5, 7, 10 and 12 Hz. In this example adding basis functions ceased to provide a significant improvement after 9–13 basis functions depending on the frequency tested.

A major shortcoming of the F-test is the fact that it does not allow one to compare non-nested basis sets. This form of model comparison generally proceeds within the Bayesian framework. In these Bayesian model comparisons, the log odds ratio implicit in the classical F-test is replaced with the more general log evidence—as approximated with variational free energy (Friston et al., 2007). Fig. 8B shows an example using the Parametric Empirical Bayes (PEB, (Friston et al., 2002)) to compare two basis sets available in SPM: Fourier and Fourier Hanning (the latter is obtained by modulating the basis set functions with a Hanning taper). For all frequencies but the lowest (5 Hz) the Fourier bases set had higher model evidence. The results for number of basis functions were comparable to those of the F-test—between 8 and 13.

It is important to use data and frequencies containing features that can be explained by the regressors in the design matrix to get meaningful results in this procedure. In principle, it would also be advisable to use independent data for basis set optimisation. However, we felt this would be unnecessary for our particular application as the GLM analysis results were not very sensitive to the choice of basis set order. Basically, the basis set should be able to fit physiologically relevant power modulations which are rather slow. An over-complete basis set is not very problematic except for unnecessary loss of degrees of freedom and slightly under-complete basis set will have an effect similar to over-smoothing the data.

Both F-test and PEB procedure as well as optimal estimation of the GLM require estimating the noise covariance matrix using restricted maximum likelihood (ReML) approach (Friston et al., 2002). For realistic time–frequency datasets the matrices involved in the computation become very large and the computation is not feasible with the current implementation in SPM8. We, therefore, only used a relatively short data segment (about 60 s long) for the basis set optimisation where ReML was used. In our other analyses for computational efficiency we used a slightly suboptimal ordinary least squares approach (or weighted least squares with pre-specified covariance matrix for artefact suppression). This does not hinder statistical inference at the 2nd level where the covariance is estimated across subjects using the standard SPM approach.

### Summary

In this section, we have illustrated the performance of the deconvolution method in relation to standard post-hoc averaging. Using simulated data, the deconvolution approach appears to finesse problems introduced by variations in the relative timing of trial-specific induced response components—illustrated here in terms of event-related synchronisation and desynchronisation. We have also touched on both classical and Bayesian model comparison that can be used, operationally, to optimise the form or number of components in the basis set used to model induced response functions to events of interest.

## An empirical demonstration

We close with a brief demonstration of the deconvolution approach by applying it to real MEG data. These analyses are simply presented to provide proof of principle that the convolution method works in practice and to indicate the sorts of results that one can obtain. In what follows we briefly describe the data and the particular comparison of interest.

A 28 year old right-handed female was asked to perform a change-of-plan paradigm (Brown and Braver, 2005; Logan and Burkell, 1986; Nachev et al., 2007) as part of a larger experiment whilst we acquired MEG data. Here we limit the data to the two relevant task blocks performed. The change-of-plan task is a variant of the stop-signal task, which was originally formulated to allow estimation of the minimum time required to stop a prepared activity—the stop-signal reaction time (SSRT) (Logan and Cowan, 1984). The subject was presented with a fixation cross (lasting 1.3–1.5 s, the duration was drawn from a uniform distribution) which, after a 200 ms pause with a blank screen, was followed by a left- or right- pointing arrow (the primary task stimulus, or ‘go signal’). The subject was asked to make a button-press with the thumb of the corresponding hand as quickly as possible. This is called the primary task. In a randomly selected 50% of trials, a change signal (a vertical red line) was presented at a variable latency (called the stimulus onset asynchrony, SOA) after the primary task stimulus, instructing the subject to cancel the primary task response (i.e. to withhold pressing the corresponding button) and switch to pressing the *opposite* button. The SOA was dynamically altered on a trial-by-trial basis according to a staircase—a correctly switched response resulted in the SOA increasing by 50 ms (making the task more difficult) whereas after a failure to change, the SOA reduced by 50 ms (alerting the subject earlier to make a change, making the task easier). This staircase procedure ensured that the probability of switching after a change signal remained ~ 50%, and so successful-switch and failed-switch conditions were equally sampled. The SOA was randomly drawn from 2 independent staircases, so that both the presence and timing of the stop-signal remained difficult for the subject to predict. Three trial-types result: go only trials (where the change-signal is not presented), successful-switch trials (where the change signal is presented and subject successfully switches) and failed-switch trials (where the change signal is presented, but the subject does not switch). Other trials, such as non-change signal trials where the left button was pressed in response to the right arrow were considered errors and discarded (not included in the model). Note that since the incorrect trials were sufficiently separated in time from the correct trials, the corresponding regressors would be orthogonal to the regressors for correct trials—were they included in the model. Thus, omitting the regressors for error trials cannot bias the estimate of our summary statistics (parameter estimates summarising induced trial-specific responses). The short data segment we used for basis set optimisation, where residuals were important, did not include erroneous trials.

Here, our aim is to isolate the induced response to a successful-switch signal. The average of epoched data, centred on successful-switch signals, is difficult to interpret because it is confounded by temporally overlapping responses to the go signal and due to the button press. In addition, attempts to use baseline correction are also difficult because of the trial-to-trial variability in the relative timing of the preceding go signal. Conventional analysis of the induced response to the go signal and button press are similarly confounded by each other and the response to the change signal.

We used the convolution model to address the above issues. MEG data were acquired at 600 Hz with a 275 channel CTF system. The data were down-sampled to 300 Hz, high-pass filtered above 0.1 Hz, and the line noise artefacts at 50 Hz and 100 Hz were removed using notch filters (5th order zero-phase Butterworth filters). We then extracted time-series data from the supplementary motor area (SMA) using a linearly constrained minimum variance (LCMV) beamformer (Van Veen et al., 1997), 0.01% regularisation and orientation in the direction of maximum power. SMA was defined by coordinates from a meta-analysis of 126 fMRI and PET studies (Mayka et al., 2006). These coordinates were transformed to subject-specific space by affine transformation based on coregistration of the subject's head to the MNI template—using fiducial markers (Litvak et al., 2011). For the conventional analysis, the time-series were epoched into 3.6 s long epochs centred on the primary task go stimulus (left and right conditions), the change stimulus (successful and failed-switch conditions) and the button press (left and right conditions). Although we were interested in the 1.5 s before and after each event, we extended the epoching window to +/− 1.8 s around the event in order to allow for both a baseline to be specified and a buffer to prevent against edge artefacts. Each trial underwent multitaper spectral analysis using the same settings used in the simulations described above (time window 0.4 s shifted in steps of 0.05 s, frequency range from 2.5 to 90 Hz in steps of 2.5 Hz, frequency resolution was set to the inverse of the time window (2.5 Hz) for up to 25 Hz, then 0.1 times the frequency for 25 to 50 Hz and then to constant 5 Hz). The square root of these data were averaged and then baseline corrected by subtracting the mean power from 1.6 s to 1 s pre-event.

For the convolution analysis, we computed continuous power for the whole recording using the same settings as above. We then specified regressors for the fixation cross, the primary task stimulus (separately for left and right arrows), and button press responses (separately for left and right responses) and the change signal (separately for successful and failed change conditions). Each regressor was a window from − 1.5 s to +1.5 s relative to the modelled event. Additionally the data and the design were filtered below 0.25 Hz.

Time–frequency plots were generated using both *post-hoc* averaging and convolution techniques and smoothed by a 7.5 Hz by 0.5 s Gaussian kernel.

### Results

**Fig. 9:**
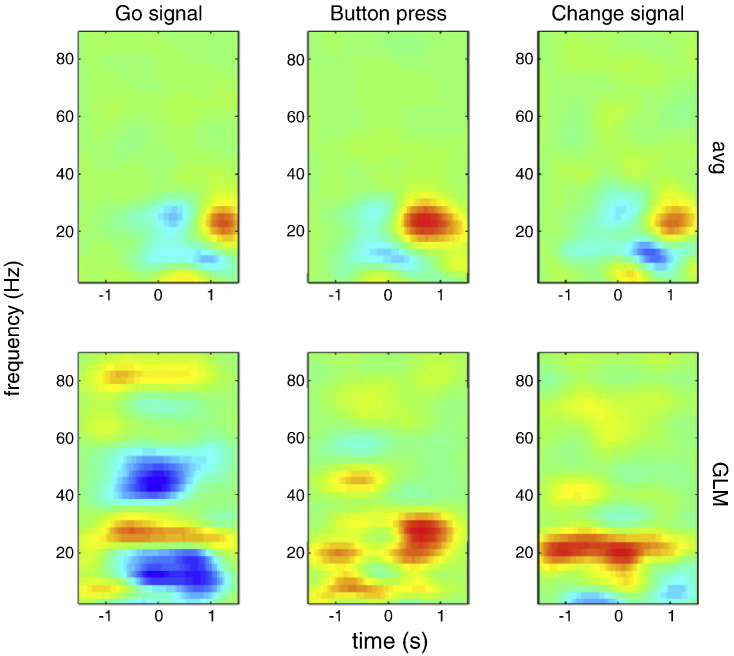
SMA activity during the change-of-plan task. Time–frequency plots for conventional averaged epochs after baseline correction (top row) and similar epochs generated using the convolution model (bottom row). The images are centred on the go signal (left), button press (centre) and change signal during a successful change response (right). The specific responses to closely overlapping events only emerged in the convolution analysis and were impossible to disambiguate from the epoched data alone.

After trials without a response and with unspecified errors (see methods) were excluded, a total 149 trials remained, with the primary task go signal being left-sided (i.e. instructing the subject to press with the left hand) in 74 and right-sided in 75 trials. In 73 trials a change signal was presented and this was successfully followed in 59% (n = 43 successful, n = 30 unsuccessful) of trials. Median reaction time was 0.546 s (simple go condition), 0.494 s (failed-change condition) and 0.718 s (successful-change condition). Median SOA for the failed-change condition was 0.350 s and for the successful-change condition 0.300 s. Fig. 9 shows the time–frequency images for *post-hoc* averaging (top row) and convolution analysis (bottom row). Reassuringly, the plots are broadly similar for both methods. Conventional analysis revealed an alpha/beta ERD starting around the time of the go signal, continuing at the time of the button press, and changing to a beta ERS immediately following the button press. However it is unclear whether this pattern was induced entirely by the go signal, or if separate components were induced by the go signal and button press. The convolution analysis revealed that the latter was the case: the go signal being associated with a clearer alpha/beta ERD (but no rebound beta ERS), and the button press being associated with a beta ERS. Conventional analysis of the successful switch condition was difficult to interpret because it was confounded, and in this case dominated, by the alpha/beta changes associated with the neighbouring go signal and button response. However, the convolution approach included these confounding events in the model, and was able to unmask an underlying increase in beta power preceding and simultaneous to a successful change signal. Therefore, in this example, the convolution model was able to disambiguate separate components of the conventional induced response, and also reveal more subtle components not visible in the conventional analysis.

## Conclusion

We have demonstrated that the use of convolution models for the analysis of M/EEG power data can overcome several inherent limitations of the established epoching method based on *post-hoc* averaging. The convolution method does not require clearly defined—non-overlapping—trials with fixed timing. This opens the way for more flexible and naturalistic experimental designs. The problem of defining a baseline period is resolved by removing slow power drifts—as an alternative to baseline correction. Finally, the convolution method can differentiate between true power changes in the context of changes in the relative latencies of different, within trial, response components. This can be crucial when an experimental factor affects the timing of compound events (e.g. the reaction time).

The General Linear Model assumes that the response can be modelled as a linear mixture of effects that are modelled by different regressors. This assumption may not be necessarily true for induced responses, which are a nonlinear function of the original signal. However, we have shown that the square root transform—that reflects the original signal amplitude—can finesse this problem and make the induced power data suitable for parametric modelling with additive noise. However—as noted by our reviewers—there may be instances where the responses to inputs or stimuli interact in a nonlinear way to produce induced responses (e.g. by changing the degree of local synchrony or phase-locking). This possibility can be addressed within the convolution framework described in this paper. This is because the general linear model can be augmented to include second order kernels, which enable one to, effectively, perform a generalised (non-linear) deconvolution using either polynomial expansions (Büchel et al., 1996, 1998) or Volterra kernels (Friston et al., 1998, 2000). This is potentially important because (as with early fMRI analyses) it becomes possible to test for nonlinear interactions among response components. In other words, to test the adequacy of linear summation assumptions (see (Friston et al., 2000) for a detailed explanation of how this is implemented formally). Relaxing the linearity assumption might also be important for making the convolution framework applicable to measures that cannot be rendered approximately linear (e.g. coherence). We are planning to address this issue in future work.

To exploit the convolution method, it is important to optimise the experimental design to reduce correlations between regressors—in other words maximise the efficiency of the design (Dale, 1999; Josephs and Henson, 1999). This might entail increasing the variability in the latencies of response components or introducing trials that preclude certain components. We recognise that this might be difficult for some experimental designs. Thus we see the convolution method not as a replacement for the averaging method but as a way to supplement it, particularly in experiments with multiple trial components that have inconstant time courses.

## Footnotes

1 In using the terms event related synchronisation and de-synchronisation, we do not necessarily imply that fluctuations in the amplitude of induced responses are mediated by changes in synchronisation but use the terms to cover any physiologically mediated changes in amplitude.
